# The impact of an 8-year mass drug administration programme on prevalence, intensity and co-infections of soil-transmitted helminthiases in Burundi

**DOI:** 10.1186/s13071-016-1794-9

**Published:** 2016-09-22

**Authors:** Giuseppina Ortu, Mohamad Assoum, Udo Wittmann, Sarah Knowles, Michelle Clements, Onésime Ndayishimiye, Maria-Gloria Basáñez, Colleen Lau, Archie Clements, Alan Fenwick, Ricardo J. Soares Magalhaes

**Affiliations:** 1 Schistosomiasis Control Initiative, Imperial College London, Department of Infectious Disease Epidemiology, School of Public Health, Faculty of Medicine (St. Mary’s Campus), Norfolk Place, London, W2 1PG UK; 2 School of Medicine, The University of Queensland, Brisbane, Australia; 3 Children’s Health and Environment Program, Child Health Research Centre, The University of Queensland, Brisbane, Australia; 4 Programme National Intégré de lutte contre les Maladies Tropicales Négligées et la Cécité (PNIMTNC) - Ministère de la Santé Publique et de la lutte contre le SIDA, Bujumbura, Burundi; 5 London Centre for Neglected Tropical Disease Research, Imperial College London, Department of Infectious Disease Epidemiology, School of Public Health, Faculty of Medicine (St. Mary’s Campus), Norfolk Place, London, W2 1PG UK; 6 Research School of Population Health, Australian National University, Canberra, Australia; 7 School of Veterinary Science, The University of Queensland (Gatton Campus), Via Warrego Highway, Gatton, QLD 4343 Australia

**Keywords:** Burundi, Soil-transmitted helminth infections, Mass drug administration, Impact, Prevalence, Intensity, Spatial dependence analysis

## Abstract

**Background:**

Soil-transmitted helminth (STH) infections are amongst the most prevalent infections in the world. Mass drug administration (MDA) programmes have become the most commonly used national interventions for endemic countries to achieve elimination. This paper aims to describe the effect of an 8-year MDA programme on the prevalence, intensity of infection and co-infection of STH in Burundi from 2007 to 2014 and critically appraise the trajectory towards STH elimination in the country.

**Results:**

Annual STH parasitological surveys (specifically, a “pilot study” from 2007 to 2011, an “extension study” from 2008 to 2011, and a “national reassessment” in 2014; *n* = 27,658 children), showed a significant drop in prevalence of infection with any STH (“pooled STH”) between baseline and 2011 in both studies, falling from 32 to 16 % in the pilot study, and from 35 to 16 % in the extension study. Most STH infections were of low intensity according to WHO classification. The national reassessment in 2014 showed that prevalence of pooled STH remained significantly below the prevalence in 2007 in both studies but there was no further decrease in STH prevalence from 2011 levels during this time. Spatial dependence analysis showed that prevalence of *Trichuris trichiura* and *Ascaris lumbricoides* had a tendency to cluster over the years, whilst only trends in spatial dependence were evident for hookworm infections. Spatial dependence fluctuated over the course of the programme for *Ascaris lumbricoides* and *Trichuris trichiura.* However, spatial trends in spatial dependence were evident in 2010 for *Ascaris lumbricoides*. Analysis of spatial clustering of intensity of infection and heavy infections revealed that the intensity changed over time for all parasites. Heavy intensity was only evident in *Ascaris lumbricoides* for 2008 and did not appear in proceeding years and other parasites.

**Conclusions:**

These results demonstrate that sustained annual MDA significantly reduced the prevalence of STH infection in school-age children but was unable to achieve elimination. Additionally, significant decline in prevalence was accompanied by a drop in spatial clustering of infection indicators across all sites from 2008. The lack of consistency in the results of the spatial dependence analysis highlights that MDA programmes can interrupt the normal transmission dynamics of STH parasites.

**Electronic supplementary material:**

The online version of this article (doi:10.1186/s13071-016-1794-9) contains supplementary material, which is available to authorized users.

## Background

Soil-transmitted helminthiases (STHs), comprising ascariasis, trichuriasis and hookworm infection are among the most common neglected tropical diseases (NTDs) affecting mainly developing countries and regions with poor sanitation [1–3]. In 2010, over 1.7 billion people were reported to be affected by these diseases globally, leading to an estimated 5 million disability-adjusted life-years (DALYs) [4]. As part of the efforts to control the morbidity associated with these diseases, routine mass administration of anthelmintic drugs has been endorsed by the World Health Organization (WHO) under its preventive chemotherapy approach [5, 6], which has been recognized as one of the most cost-effective public health strategies to reduce the disease burden of these infections [7]. In Burundi, STH infections have long been recognised as a public health problem [8–10] and this paper discusses the impact of deworming campaigns on the burden of worm infections in Burundi between 2007 and 2014.

Burundi is a small landlocked country in eastern Africa, characterized by three main ecological zones: lakeside, hills and plateau. The population (approximately 10.6 million inhabitants), lives predominantly in rural areas, and makes a living on agriculture [11]. The Gross National Income per capita has been estimated as $270 in 2014; however, it was reported as $160 in 2007 [11]. Almost half of the population is below the age of 15, with a probability of 81.7 per 1000 that a new born baby will die before reaching age five, and with a life expectancy at birth of 57 years [11]. Children net enrolment rate in primary schools is 95.4 %, making primary schools an effective channel to deliver health services specifically targeting this age group.

In 2007, key partners and funders came together in collaboration with the Ministry of Health of Burundi to establish an integrated control programme to address NTDs [12]. As a result, in May 2007, STH infections and schistosomiasis were mapped across the entire country and results demonstrated that whereas urinary schistosomiasis was not endemic in the country, *Schistosoma mansoni* was endemic in some areas (especially along Lake Tanganyika) and STHs were highly prevalent nationwide. These results highlighted that interventions against STH infections were critically needed [13]. The findings of this study provided the basis for rolling out an integrated preventive chemotherapy programme throughout the country.

The national deworming programme covering the whole country was first launched in mid-2007, and included the delivery of albendazole (ALB) twice a year to children aged 5 to 14 years and pregnant women in their second and third trimester [12]. This drug delivery was done through primary schools, health centres and mobile clinics to reach the target population. Along this programme, Burundi was already providing mebendazole (MBZ) to children under 5 years of age within the Mother and Child Health Week (MCHW) campaigns (twice a year, coupled with the national deworming campaigns), and ALB (or sometimes MBZ, based on drug availability), to adults (for treatment of STHs, twice a year, 3–4 months apart from deworming and MCHW campaigns) within the national onchocerciasis programme in areas endemic for this disease (where annual ivermectin was distributed). Specifically, as part of the national deworming programme, a monitoring and evaluation (M&E) strategy was developed to assess the impact of treatment during the first 5 years (2007–2011). This involved performing annual parasitological surveys at a set of primary school sentinel sites. Epidemiological M&E surveys were stopped in 2011 with the completion of the national programme’s first phase of intervention, though between 2011 and 2014 ALB and MBZ treatment continued routinely, and via using the same delivery channels and treatment frequency. Later in mid-2014, with the support of the Schistosomiasis Control Initiative (SCI) and the Schistosomiasis Consortium for Operational Research and Evaluation (SCORE), a national STH re-assessment was performed to evaluate whether further STH treatment was needed.

Previous studies have demonstrated significant population-level benefits of regular deworming in terms of declining prevalence of infection [14], intensity of infection [15] and co-infections [16, 17], co-infections being defined here as being infected with more than one of the STH parasites. However, very few studies have looked at the long-term (> 5 years) nationwide effect of deworming on a range of infection indicators. Fewer studies still have attempted to understand the impact on spatial heterogeneity in these indicators as a result of a multi-year MDA. By analysing spatial heterogeneity in infection prevalence, intensity of infection categories, and co-infections, a clearer picture of the relationships between infection, the impact of the intervention, and changing patterns of spatial distribution may become evident. These results would enable the development of more efficient treatment protocols as the MDA programme progresses towards the last stages. For instance, increased clustering of infections may indicate the existence of residual transmission hotspots where more frequent treatments and other interventions such as improved sanitation and WASH protocols may be necessary.

In this study we aim to document the epidemiological impact of the 8-year (2007–2014) MDA programme in Burundi on the prevalence and intensity of STH infection, intensity of infection and co-infections, as well as spatial heterogeneity in infection indicators, by analysing STH infection data from both the nationwide M&E impact study and the 2014 re-assessment survey. Although intestinal schistosomiasis as well weight and height data were also recorded during the study period, results have not been included in this paper and will be reported elsewhere.

## Methods

### Parasitological surveys

**Fig. 1:**
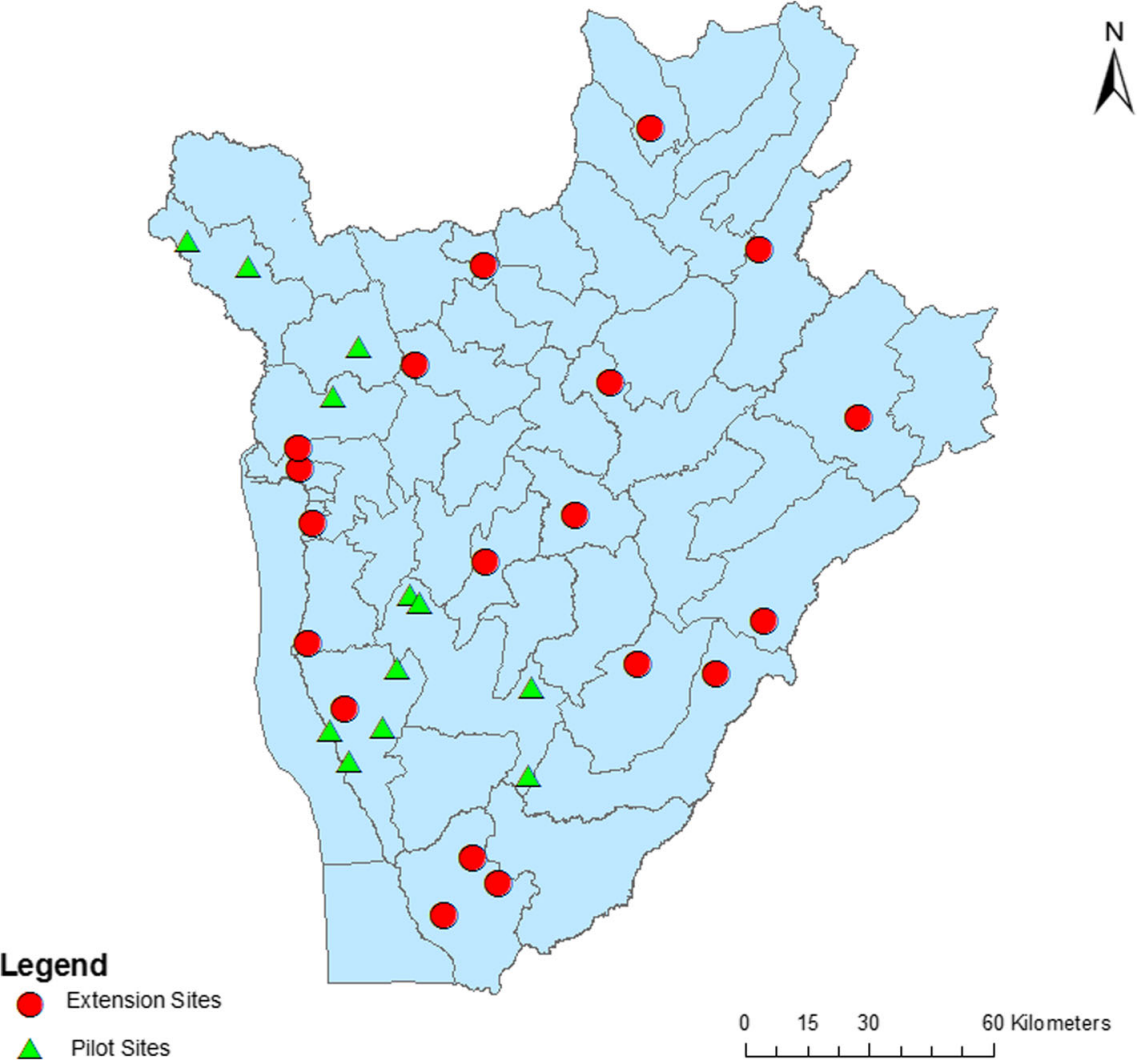
Map indicating the locations of pilot and extension studies sites in Burundi

In 2007, a total of 12 schools were selected and surveyed as sentinel sites for monitoring programmatic impact on STH (“pilot schools”), in three provinces where the programme began to be rolled out (Bururi, Bubanza and Cibitoke) [12]. In 2008, the programme was expanded to cover the entire country and 19 additional schools were selected across the remaining provinces (“extension schools”). During the nationwide STH re-assessment survey conducted in 2014, all pilot schools and 14 of the 19 extension schools were surveyed to evaluate the prevalence and intensity of STHs after 7 years of routine MDA. Figure 1 shows the locations of the schools included in each survey.

Annual parasitological surveys were conducted across all schools, with the exception of extension schools in 2010, when surveys were not possible due to political instability in parts of the country. Annual surveys were conducted approximately 1 month before each round of ALB/MBZ administration (surveys were conducted in April/May with drug distribution in June) to ensure that all children who tested positive in surveys received timely treatment through the national programme. At each pilot school, a cohort of 200 pupils from grades 1 and 2 and a cross-sectional sample of 100 pupils from grade 6 were recruited at baseline in 2007; 15.1 % of these grade 1 and 2 were followed up each year until 2011 (see Additional file 1: Table S1, for details of cohort follow-up). In each follow-up year, between 140 and 216 additional pupils from grades 1 and 6 in each school were sampled. In 2011, no cross-sectional sample of grade 6 pupils was selected.

At each extension school, a cohort of 200 pupils from grades 1 and 2 was recruited at baseline in 2008, complemented by a cross-sectional sample of 100 grade 6 pupils; 34.2 % of these grade 1 and 2 pupils were followed up each year of the study until 2011. In 2009, between 110 and 200 additional pupils per school were sampled from grade 1 and grade 6 pupils. In 2010 surveys were not possible in 18 sites out of the 31 due to political instability in parts of the country. In 2011 between 255 and 300 pupils per school were newly recruited. All surveyed individuals were under the age of 23 years, with 97 and 92 % being between 5 and 16 years old at pilot and extension schools, respectively.

In each school surveyed for the national epidemiological re-evaluation of STH in 2014, 50 pupils between the age of 12 and 16 were recruited, with the exception of one pilot study school where 100 pupils were recruited; here we include only all pilot schools and 14 of the 19 extension schools. For all surveys, approximately equal number of boys and girls were recruited.

### Parasitological examination

In all surveys, hookworm*, Ascaris lumbricoides* and *Trichuris trichiura* infections were assessed by examination of faecal samples via the Kato-Katz method. One faecal sample was taken per child, and two slides were prepared. Parasite eggs were counted on both slides by two different microscopists and read within 2 h of preparation to avoid degeneration of hookworm eggs. An estimate of intensity of infection for each species in eggs per gram of faeces was obtained by multiplying the mean number of eggs found across both slides by 24. A child was classified as being infected with a particular parasite if at least one egg was counted, classified as being infected with any STH (“pooled STH”) if at least one egg of any species was detected, and classified as being multiply infected if at least one egg from two or more species was detected. Intensity of infection was categorised based on WHO guidelines [5] (see Tables 1 and 2). Other data collected were observations of the latrine status and source of water in each school. All data were collected on case report forms completed in the field by the team, double-entered onto a MS Access database and any discrepancies resolved.

**Table 1:** Characteristics of study participants and mean prevalence estimated from the raw data of STH over five years of monitoring twelve pilot sentinel schools in Burundi. The last column contains data collected for a mapping survey in 2014 at the same schools. Added in parentheses are the standard errors for the raw mean prevalence $\left(\sqrt{\frac{\widehat{p}\left(1-\widehat{p}\right)}{n}}\right)$ and the raw intensity means $\left(\frac{\widehat{sd}}{\sqrt{n}}\right)$, respectively

Year	2007	2008	2009	2010	2011	2014
Number of participants	3,616	3,476	4,944	5,062	4,680	650
Mean age	10.81	10.66	10.6	10.84	9.93	13.19
Gender ratio (F:M)	0.53	0.51	0.51	0.52	0.52	0.5
Raw mean prevalence						
Pooled STH	0.32 (0.008)	0.19 (0.007)	0.27 (0.006)	0.17 (0.005)	0.16 (0.005)	0.18 (0.015)
*Ascaris*	0.14 (0.006)	0.11 (0.005)	0.18 (0.005)	0.10 (0.004)	0.11 (0.005)	0.12 (0.013)
*Trichuris*	0.03 (0.003)	0.02 (0.002)	0.04 (0.003)	0.02 (0.002)	0.02 (0.002)	0.03 (0.007)
Hookworm	0.18 (0.006)	0.07 (0.004)	0.08 (0.004)	0.06 (0.003)	0.04 (0.003)	0.04 (0.008)
Raw mean intensity of infected pupils only						
*Ascaris*	2,667 (397)	3,624 (624)	793 (66)	2,065 (147)	1,970 (176)	1,831 (418)
*Trichuris*	177 (41)	157 (52)	112 (24)	143 (33)	91 (20)	70 (23)
Hookworm	111 (15)	177 (28)	85 (6)	224 (51)	104 (9)	282 (151)
Intensity of Infection (uninfected/light/moderate/heavy): *n* (%)						
*Ascaris*	3,109/437/62/1	3,075/338/54/4	4,063/852/27/0	4,542/456/57/0	4,159/452/60/0	575/61/14/0
	(86/12/2/0)	(89/10/2/0)	(82/17/1/0)	(90/9/1/0)	(89/10/1/0)	(88/9/2/0)
*Trichuris*	3,488/116/4/0	3,412/62/1/0	4,728/211/2/0	4,969/85/1/0	4,578/91/2/0	629/21/0/0
	(97/3/0/0)	(98/2/0/0)	(96/4/0/0)	(98/2/0/0)	(98/2/0/0)	97/3/0/0
Hookworm	2,974/633/1/1	3,235/236/4/1	4,566/375/0/0	4,737/313/3/2	4,489/182/0/0	622/27/0/1
	(82/18/0/0)	(93/7/0/0)	(92/8/0/0)	(94/6/0/0)	(96/4/0/0)	(96/4/0/0)

**Table 2:** Characteristics of study participants and mean prevalence estimated from the raw data of STH over four years of monitoring in 19 extension sentinel sites in Burundi. The last column contains data collected for a mapping survey in 2014 at 14 of the 19 extension schools. Added in parentheses are the standard errors for the raw mean prevalence $\left(\sqrt{\frac{\widehat{p}\left(1-\widehat{p}\right)}{n}}\right)$ and the raw intensity means $\left(\frac{\widehat{sd}}{\sqrt{n}}\right)$, respectively

Year	2008	2009	2011	2014
Number of participants	5,700	6,378	8,869	700
Mean age	10.98	10.48	11.03	13.33
Gender ratio (F:M)	0.50	0.50	0.52	0.50
Raw mean prevalence				
Pooled STH	0.35 (0.006)	0.26 (0.005)	0.16 (0.004)	0.20 (0.015)
*Ascaris*	0.20 (0.005)	0.10 (0.004)	0.09 (0.003)	0.10 (0.011)
*Trichuris*	0.10 (0.004)	0.10 (0.004)	0.04 (0.002)	0.03 (0.006)
Hookworm	0.14 (0.005)	0.09 (0.004)	0.05 (0.002)	0.08 (0.010)
Raw mean intensity of infected pupils only				
*Ascaris*	2,081 (178)	1,147 (191)	1,581 (138)	1,084 (258)
*Trichuris*	122 (9)	106 (12)	86 (6)	45 (6)
Hookworm	100 (11)	85 (6)	120 (13)	151 (36)
Intensity of infection (uninfected/light/moderate/heavy): *n* (%)				
*Ascaris*	4,581/1,002/114/3	5,702/637/30/1	8,059/732/50/1	628/69/3/0
	(80/18/2/0)	(90/10/0/0)	(91/8/1/0)	(90/10/0/0)
*Trichuris*	5,150/542/6/0	5,736/628/6/0	8,478/362/2/0	682/18/0/0
	(90/10/0/0)	(90/10/0/0)	(96/4/0/0)	(97/3/0/0)
Hookworm	4,907/790/0/1	5,823/546/1/0	8,425/412/4/0	645/55/0/0
	(86/14/0/0)	(91/9/0/0)	(95/5/0/0)	(92/8/0/0)

Co-infection profiles were drawn to help to identify the distribution of the prevalence of infections based on all possible co-infection groupings. The groupings identified were: mono-infection; *A. lumbricoides* and *T. trichiura* co-infection; *A. lumbricoides* and hookworm co-infection; *T. trichiura* and hookworm co-infection, and finally *A. lumbricoides*, *T. trichiura* and hookworm co-infections. Table 2 summarises the prevalence of all co-infection combinations from 2007 to 2014.

### Temporal changes in infection indicators

To assess overall population-level programmatic impact on the prevalence of STH, we used two sets of models: one for all pilot schools and one for all extension schools. Using the *lme4* [18] package in R, binomial generalized linear mixed models (GLMMs) with a logit link were used, with infection presence/absence as the response variable, both for individual parasite species and pooled STH. We analysed all available data regardless of follow-up status [19]. McFadden pseudo-*R*^2^ [20] and a combination of marginal and conditional pseudo-*R*^2^ values [21] were used to assess the goodness-of-fit of the models. The McFadden pseudo-*R*^2^ measures the level of improvement of the log-likelihood of a model over the log-likelihood of the intercept model [20]; values of 0.2 and above are considered to indicate a very good fit [22]. Marginal and conditional pseudo-*R*^2^ values quantify the proportion of variance on the latent scale explained by the fixed effects components or the fixed and random effects components, respectively, with higher values being preferable. To examine whether there was statistical evidence for prevalence changing over time differentially among schools, we examined a standard set of models for each response variable. All models included the same set of fixed effects: year (as a factor), age (mean-centred and divided by the estimate of its standard deviation) as well as its associated quadratic (to allow for potential non-linearity in any age effect) and gender. School and pupil identity were included as random factors to account for similarity among children at the same school and repeated samples from individual children across the longitudinal study respectively, and a random interaction between year and school was included to account for the variation in response between the schools [23]. The prevalence of *T. trichiura* in the pilot study was very low (≤ 4 % in all years) and models including this variable did not converge; hence this species was excluded from the analyses.

Changes in prevalence of multiple infections were examined using two types of models, first including all pupils, and secondly including only those pupils infected with at least one STH. The model including all pupils was used to enable direct comparison with the prevalence models presented previously, and adopted the same fixed and random effects as the models above. The model including only those pupils infected with at least one STH enabled us to investigate whether intensity of infection with a single species was associated with multiple infections; including non-infected pupils in this model would mean that the response of no multiple infection could be completely predicted from knowledge of the fixed effect of intensity of infection. The model used the same fixed effects structure as described previously (excluding the quadratic age term as the model would not converge with this included) with additional fixed effects of egg counts per gram of hookworm, *T. trichiura*, and *A. lumbricoides* (each mean-centred and divided by the estimate of its standard deviation). School identity and pupil identity were included as random effects; models that included a year by school interaction did not converge and consequently this interaction was excluded from the model.

Intensity of infection was generally low throughout the study period. Although analyses of intensity of infection (egg counts per gram) were attempted, due to a large number of zero egg counts and extreme variances, models either did not converge or showed a bad fit, and intensity models were not pursued further. Since no heavy infections were detected, we could not model the prevalence of heavy infections. However, we modelled the prevalence of infections that were at least of moderate intensity, using the same predictors and model structure as described above. Only the model for *A. lumbricoides* for extension schools converged.

Data cleaning and handling were carried out in SAS v9.1.3 and R version 3.0.2 [24], and analyses were performed in R version 3.1.2.

### Assessment of spatial clustering in infection indicators

Semivariograms were used to analyse spatial dependence in STH indicators, and how this changed over time [25, 26]. Semivariograms allow for the quantification of spatial cluster size and the tendency for geographical clustering within a region by estimating three parameters: the partial sill, the nugget and the range. The partial sill refers to the variance in the infection indicators between pairs of survey locations that is due to factors associated with geographical location. The nugget refers to the variance in the infection indicators between pairs of survey locations that is due to measurement error. The range is the distance up to which observations are considered dependent and is an estimate of the average size of the clusters. To estimate the proportion of residual variation that was spatially structured, we divided the partial sill by the sum of the partial sill and the nugget. Empirical semivariograms were constructed by combining data from pilot and extension schools for each parasite species, using raw (observed) data on infection prevalence, prevalence of intensity profiles (based on WHO guidelines [27, 28]) and prevalence of co-infections at each survey locations. We did not analyse prevalence of high intensity infections for *A. lumbricoides* due to the very limited number of children exhibiting this infection pattern. For the same reason, prevalence of moderate and high intensity infections for *T. trichiura* and hookworm were excluded from further analyses.

The semivariograms for prevalence of co-infection were also prepared based on the most prevalent co-infection pattern, i.e. *A. lumbricoides* and *T. trichiura*, for years 2007 to 2011 (Additional file 1: Figure S1). On initial observation, in 2014, only five individuals were reported with this co-infection pattern. Other co-infection combinations were equally rare, with only seven individuals with *A. lumbricoides* and hookworm co-infection; two cases of hookworm and *T. trichiura* co-infections and no cases of a triple infection evident in this year. As such, because of the low level of co-infections, no further analysis was pursued. Table 3 shows the temporal breakdown of co-infection combinations. Empirical semivariograms were constructed using data from all years combined as well as for each year separately. The values of the partial sill, nugget and range were estimated by fitting Weighted Least Squares to the empirical semivariogram. If the line of best fit levels out within the empirical semivariogram, then the infection indicator is classified as clustered. If the line of the best fit appears to be continually increasing, then the infection indicator is classified as depicting a spatial trend. Finally, if it is not possible to fit a (statistically meaningful) line through the empirical semivariogram, then it is considered that there is no spatial clustering in the infection indicator. We elected not to use fixed effects in our models in order to capture the natural trends in the data. All analyses were conducted using the *geoR* package v2·14·1 in R statistical software [29, 30].

**Table 3:** Co-infection prevalence from 2007 to 2011, and mapping study 2014

Year	Not infected (%)	Mono-infected (%)	AT (%)	AH (%)	TH (%)	ATH (%)
2007	68.04	29.09	1.12	1.43	0.22	0.08
2008	71.01	24.14	2.72	1.03	0.50	0.60
2009	72.96	24.43	1.48	0.57	0.42	0.14
2010	82.61	16.66	0.55	0.12	0.04	0.02
2011	84.19	14.24	1.15	0.24	0.16	0.03
2014	81.11	17.85	0.37	0.52	0.15	0.00

Abbreviations: A, *Ascaris*; T, *Trichuris*; H, hookworm

## Results

### Data for analysis

Sample sizes and characteristics of the children recruited at baseline and surveyed at each time-point at the two sets of sentinel sites are shown in Tables 1 and 2. A total of 42,725 epidemiological assessments were conducted throughout the 5 years across all sentinel sites (21,778 for the pilot study and 20,947 for the extension study); the percentage of individuals recruited each year and the nested cohort followed throughout the years can be found in Additional file 1: Table S1. Mean participant age was relatively constant across sentinel sites, ranging from 9.93 to 10.84 years between 2007 and 2011 for the pilot study and ranging between 10.48 and 11.03 from 2008 to 2011 for the extension study. In the 2014 re-evaluation surveys, the mean age was 13.19 and 13.33 years for the pilot and the extension schools respectively.

### Changes in prevalence of infection and co-infection over time

#### Baseline prevalence of STH

**Fig. 2:**
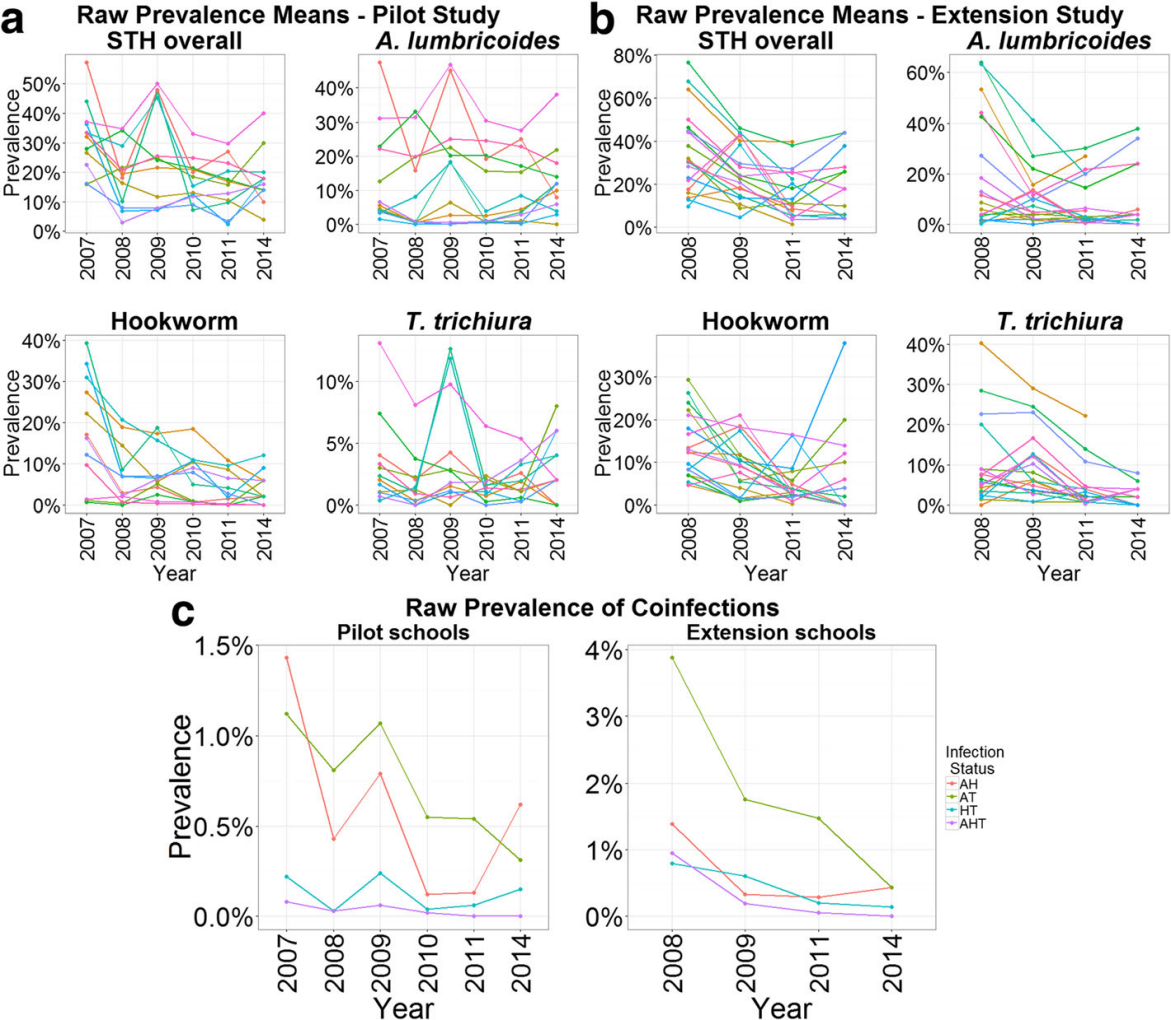
Temporal changes (2007 to 2014) in the (raw) prevalence of soil-transmitted helminthiases (STH) in Burundi. Temporal changes in the raw prevalence of individual and pooled STH parasite species, as well as co-infection, at **a** pilot and **b** extension study schools. In **a** and **b**, each line represents an individual school. In **c**, each line represents one of the co-infection types analysed: *Ascaris* and Hookworm (AH), *Ascaris* and *Trichuris* (AT), Hookworm and *Trichuris* (HT), and infection with all three STH species (AHT)

Raw prevalence values in each year of the study for each STH species individually and pooled are shown in Tables 1 and 2, and in Fig. 2. At baseline, hookworm and *A. lumbricoides* were the most prevalent STH species. Baseline prevalence levels of *A. lumbricoides* and hookworm were 14 and 18 %, respectively, in the pilot schools and 20 and 14 %, respectively, in the extension schools. The overall baseline prevalence of *T. trichiura* was below 10 % (Table 1).

#### Temporal changes in pooled STH prevalence

Results of models analysing pooled STH prevalence are shown in Tables 4, 5 and Additional file 1: Figures S2 and S3 illustrate these models with prevalence plotted separately for each school. The pseudo-*R*^2^ values for both models were low, especially for the pilot schools models. In both pilot and extension schools, STH prevalence declined significantly between baseline and 2011, and also between baseline and 2014 (Tables 4 and 5). In both sets of sentinel sites, coefficients comparing baseline with 2014 were less in absolute size than those comparing baseline with 2011, implying that STH prevalence did not decrease further between 2011 and 2014. Boys were significantly more likely to harbour STH infections than girls in extension schools, and age had a significant influence on the risk of infection. In the pilot schools the risk of infection increased with age while in the extension schools it was maximal at the mean age and decreased for ages below and above the average age.

**Table 4:** Predictors of infection with STH overall, *Ascaris lumbricoides* and hookworm, across pilot study sentinel sites in Burundi from 2007 to 2011 combined with prevalence data obtained by a mapping survey in 2014 from the same schools. Results shown are from binomial mixed models. Age was standardised (mean centred and divided by the estimated standard deviation)

		Pooled STH			*Ascaris lumbricoides*			Hookworm		
		*n* = 22,341 observations pseudo-*R* ^2^_MF_ = 0.057; *R* ^2^_marg._ = 0.037; *R* ^2^_cond._ = 0.21			*n* = 22,347 observations pseudo-*R* ^2^_MF_ = 0.17; *R* ^2^_marg._ = 0.028; *R* ^2^_cond._ = 0.40			*n* = 22,351 observations pseudo-*R* ^2^_MF_ = 0.13; *R* ^2^_marg._ = 0.079; *R* ^2^_cond._ = 0.48		
Fixed effects	Category	Parameter (SE)	Adjusted odds ratio (95 % CI)	*P*	Parameter (SE)	Adjusted odds ratio (95 % CI)	*P*	Parameter (SE)	Adjusted odds ratio (95 % CI)	*P*
(Intercept)		-0.90 (0.17)	0.41 (0.29–0.56)	***	-2.5 (0.36)	0.08 (0.04–0.17)	***	-2.20 (0.44)	0.11 (0.05–0.26)	***
Year	2008	-0.90 (0.26)	0.4 (0.24–0.67)	***	-1.05 (0.4)	0.35 (0.16–0.76)	**	-1.29 (0.28)	0.28 (0.16–0.47)	***
	2009	-0.43 (0.23)	0.65 (0.41–1.01)	ns	-0.24 (0.45)	0.79 (0.33–1.89)	ns	-0.87 (0.35)	0.42 (0.21–0.82)	*
	2010	-0.90 (0.20)	0.41 (0.27–0.61)	***	-0.84 (0.26)	0.43 (0.26–0.72)	**	-1.36 (0.34)	0.26 (0.13–0.5)	***
	2011	-1.14 (0.23)	0.32 (0.2–0.5)	***	-0.48 (0.2)	0.62 (0.41–0.92)	*	-2.26 (0.38)	0.10 (0.05–0.22)	***
	2014	-0.85 (0.25)	0.43 (0.26–0.71)	***	0.05 (0.31)	1.05 (0.57–1.93)	ns	-1.48 (0.5)	0.23 (0.09–0.6)	**
Age		-0.07 (0.019)	0.94 (0.9–0.97)	***	-0.12 (0.025)	0.89 (0.85–0.93)	***	0.02 (0.028)	1.02 (0.97–1.08)	ns
Age^2		-0.01 (0.017)	0.99 (0.95–1.02)	ns	-0.03 (0.023)	0.97 (0.93–1.01)	ns	-0.02 (0.025)	0.98 (0.94–1.03)	ns
Sex (male)		0.09 (0.037)	1.09 (1.01–1.17)	*	0.08 (0.048)	1.08 (0.98–1.19)	ns	0.09 (0.053)	1.09 (0.98–1.21)	ns
Random Effects		Variance	SD		Variance	SD		Variance	SD	
Pupil (Intercept)		0.43	0.66	***	0.56	0.75	***	0.33	0.57	***
School (Intercept)		0.31	0.55		1.50	1.23		2.20	1.50	
Year 2008		0.76	0.87		1.60	1.25		0.63	0.79	
Year 2009		0.59	0.77		2.20	1.48		1.3	1.14	
Year 2010		0.46	0.68		0.66	0.81		1.2	1.10	
Year 2011		0.59	0.77		0.36	0.60		1.1	1.05	
Year 2014		0.60	0.78		0.84	0.92		1.8	1.35	
Random Slope year				***			***			***

*Abbreviations*: *ns* not significant (*P* > 0.05); *SD* standard deviation; *SE* standard error

*P ≤ 0.05; **P ≤ 0.01; ***P ≤ 0.001

**Table 5:** Predictors of infection with pooled STH species, *Ascaris lumbricoides,* hookworm, and *Trichuris trichiura* across 19 extension study sentinel sites in Burundi from 2008 to 2011 combined with prevalence data obtained by a re-evaluation survey in 2014 from 14 of the 19 extension schools. Results shown are from binomial mixed models. Age had been standardised (mean centred and divided by estimated standard deviation)

		Pooled STH			*Ascaris lumbricoides*		
		*n* = 20,871 observations, pseudo-*R* ^2^_MF_ = 0.13, *R* ^2^_marg._ = 0.064; *R* ^2^_cond._ = 0.31			*n* = 20,879 observations, pseudo-*R* ^2^_MF_ = 0.22, *R* ^2^_marg._ = 0.024; *R* ^2^_cond._ = 0.50		
Fixed Effects		Parameter (SE)	Adjusted odds ratio (95 % CI)	*P*	Parameter (SE)	Adjusted odds ratio (95 % CI)	*P*
(Intercept)		-0.75 (0.22)	0.47 (0.31–0.73)	***	-2.22 (0.39)	0.11 (0.05–0.23)	***
Year	2009	-0.54 (0.21)	0.58 (0.39–0.88)	*	-0.52 (0.31)	0.59 (0.32–1.09)	ns
	2011	-1.30 (0.17)	0.27 (0.2–0.38)	***	-0.92 (0.22)	0.40 (0.26–0.62)	***
	2014	-1.01 (0.21)	0.36 (0.24–0.55)	***	-0.71 (0.32)	0.49 (0.26–0.93)	*
Age		-0.02 (0.02)	0.98 (0.94–1.02)	ns	-0.08 (0.026)	0.92 (0.87–0.97)	**
Age^2		-0.06 (0.017)	0.94 (0.91–0.98)	***	-0.04 (0.023)	0.96 (0.92–1.01)	ns
Sex (male)		0.12 (0.037)	1.13 (1.05–1.22)	***	0.11 (0.048)	1.12 (1.02–1.23)	*
Random Effects		Variance	SD		Variance	SD	
Pupil (Intercept)		0.24	0.49	***	0.26	0.51	***
School (Intercept)		0.90	0.95		2.82	1.68	
Year 2009		0.78	0.89		1.65	1.28	
Year 2011		0.47	0.69		0.81	0.90	
Year 2014		0.44	0.67		1.08	1.04	
Random Slope year				***			***
		Hookworm			*Trichuris trichiura*		
		*n* = 20,876 observations, pseudo-R^2^_MF_ = 0.096, *R* ^2^_marg._ = 0.082; *R* ^2^_cond._ = 0.22			*n* = 20,877 observations, pseudo-*R* ^2^_MF_ = 0.15, *R* ^2^_marg._ = 0.058; *R* ^2^_cond._ = 0.42		
Fixed Effects		Parameter (SE)	Adjusted odds ratio (95 % CI)	*P*	Parameter (SE)	Adjusted odds ratio (95 % CI)	*P*
(Intercept)		-2.05 (0.16)	0.13 (0.09–0.18)	***	-2.94 (0.28)	0.05 (0.03–0.09)	***
Year	2009	-0.79 (0.21)	0.45 (0.3–0.68)	***	0.08 (0.23)	1.09 (0.70–1.69)	ns
	2011	-1.39 (0.18)	0.25 (0.17–0.36)	***	-1.07 (0.18)	0.34 (0.24–0.49)	***
	2014	-1.60 (0.49)	0.20 (0.08–0.53)	**	-1.21 (0.33)	0.30 (0.16–0.56)	***
Age		0.05 (0.029)	1.05 (1–1.12)	ns	-0.02 (0.031)	0.98 (0.92–1.04)	ns
Age^2		-0.03 (0.025)	0.97 (0.92–1.01)	ns	-0.1 (0.028)	0.90 (0.85–0.95)	***
Sex (male)		0.12 (0.052)	1.13 (1.02–1.25)	*	0.09 (0.058)	1.09 (0.98–1.22)	ns
Random Effects		Variance	SD		Variance	SD	
Pupil (Intercept)		0.16	0.40	ns	0.66	0.81	***
School (Intercept)		0.41	0.64		1.40	1.18	
Year 2009		0.70	0.84		0.82	0.90	
Year 2011		0.52	0.72		0.39	0.63	
Year 2014		1.96	1.4		0.28	0.53	
Random Slope year				***			***

*Abbreviations*: *ns* not significant (*P* > 0.05); *SD* standard deviation; *SE* standard error

*P ≤ 0.05; **P ≤ 0.01; ***P ≤ 0.001

#### Temporal changes in individual species prevalence

In 2011, following repeated annual treatment, the prevalence of all individual STH species was significantly less than at baseline in both pilot and extension schools (Tables 4 and 5). In both pilot and extension schools, prevalence reductions from baseline to 2011 were strongest for hookworm (Adjusted Odds Ratio (AOR) = 0.10 in pilot schools and 0.25 in extension schools, *P* < 0.001 for both) and weakest for *A. lumbricoides* (AOR = 0.62, *P* = 0.017 for pilot schools and AOR = 0.40, *P* < 0.001 for extension schools). Prevalence reduction of *T. trichiura* in extension schools was intermediate between hookworm and *A. lumbricoides* (AOR = 0.34, *P* < 0.001), and the *T. trichiura* models in the pilot studies did not converge.

Patterns of change in individual species between baseline and 2014, were similar to between baseline and 2011 but less pronounced. In both pilot and extension schools again hookworm showed the strongest decline from baseline. However, the magnitude of reduction in pilot schools in 2014 (AOR = 0.23, *P* = 0.003) compared to baseline was less than in 2011, but in extension schools was greater than 2011 (AOR = 0.20, *P* = 0.0012). For *A. lumbricoides*, in the pilot schools there was no evidence of reduction in prevalence between baseline and 2014 (AOR = 1.05, *P* = 0.87) and the extension schools showed less reduction in prevalence from baseline compared to 2011 (AOR = 0.49, *P* = 0.028), implying that prevalence did not decrease further between 2011 and 2014 (Tables 4 and 5). Similar to *A. lumbricoides* in the extension schools, *T. trichiura* prevalence in 2014 was significantly lower than at baseline but the estimate of the reduction was very slightly less than in 2011 (AOR = 0.3, *P* < 0.001)*.*

Prevalence of *A. lumbricoides* decreased with age for both the pilot and the extension schools while the maximum prevalence of infection of *T. trichiura* in the extension schools was reached at approximately 11 years of age (Tables 4 and 5). Age was not statistically significant factor for hookworm in both models (Tables 4 and 5).

Sex did not have a significant effect on the risk of infection in the pilot schools. In the extension schools the risk of infection was greater for boys for hookworm (*P* = 0.019) and *A. lumbricoides* (*P* = 0.021) although pseudo-*R*^2^ values for the hookworm model were small.

For all models, the large difference between the pseudo-*R*^2^ based on the fixed effects only (marginal pseudo-*R*^2^) and based on the fixed and random effects (the conditional pseudo-*R*^2^) suggested that a large portion of the variation in the data was explained by differences between schools and individual pupils. Furthermore, the magnitude and significance of the year by school interaction (*P*-value less than 0.001 for all models), showed that temporal changes in prevalence differed notably across schools. In all single-species models, the probability of infection was influenced more significantly by school-level than individual-level factors (Tables 4 and 5). The variance explained by school was greatest for *A. lumbricoides,* especially in the pilot study.

#### Prevalence of multiple-infections

The prevalence of multiple infection was generally low (Table 3, Fig. 2c), with the highest multiple infection prevalence seen in 2008, with 4.85 % individuals infected with two or more species. For the prevalence of multiple infections based on all children, to enable comparison with previous analyses, only models for extension schools converged. The model based on all pupils (infected and uninfected) indicated a steady decline in the prevalence of multiple infections from baseline with the greatest decline in 2011 (Table 6). When analysis was restricted to only children infected with at least one species, to enable intensity of infection of individual species to be included in the model, again only the extension study models converged. The adjusted odds ratios for *T. trichiura* was 3.12, i.e. more than twice the adjusted odds ratios for *A. lumbricoides* or hookworm (Table 7), indicating that infection with *T. trichiura* was associated with a greater probability of multiple infection than the other two infection types.

**Table 6:** Predictors for the presence of multiple infections across all 19 extension study sentinel sites in Burundi combined with the data obtained by a mapping survey in 2014 from 14 of the 19 extension schools. All pupils (infected and uninfected, *n* = 20,871 observations) were included in the data. Results are from a binomial mixed model, age had been mean-centred and divided by the estimate of its standard deviation (SD). The pseudo-*R* ^2^ values for this model were pseudo-*R* ^2^_MF_ = 0.196; *R* ^2^_marg_ = 0.079; *R* ^2^_cond._ = 0.479

Fixed effects	Category	Parameter	Adjusted odds ratio (CI)	*P*
(Intercept)		-3.61 (0.35)	0.03 (0.01–0.05)	< 0.001
Year	2009	-0.57 (0.21)	0.57 (0.37–0.86)	0.008
	2011	-1.59 (0.21)	0.2 (0.13–0.31)	< 0.001
	2014	-1.88 (0.72)	0.15 (0.04–0.62)	0.009
Sex (male)		0.17 (0.078)	1.18 (1.01–1.37)	0.033
Age		-0.03 (0.044)	0.97 (0.89–1.06)	0.529
Age^2		-0.09 (0.039)	0.91 (0.84–0.98)	0.016
Random effects		Variance	SD	*P*
Pupil (Intercept)		0.55	0.74	< 0.001
School (Intercept)		2.00	1.40	
Year 2009		0.50	0.71	
Year 2011		0.30	0.55	
Year 2014		2.00	1.40	
Random slope year				< 0.001

**Table 7:** Predictors for the presence of multiple infections across all extension study sentinel sites in Burundi combined with the data obtained by a mapping survey in 2014 from 14 of the 19 extension schools. Only data from infected pupils (*n* = 5,027 observations) was included. Results are from a binomial mixed model. Age and the egg counts for *Ascaris lumbricoides*, hookworm, and *Trichuris trichiura* in epg had been mean-centred and divided by the estimates of their standard deviations. The pseudo-*R* ^2^ values for this model were pseudo *R* ^2^_MF_ = 0.186; *R* ^2^_marg._ = 0.300; *R* ^2^_cond._ = 0.402

Fixed effects	Category	Parameter	Adjusted odds ratio (CI)	*P*
(Intercept)		-2.06 (0.2)	0.13 (0.09–0.19)	< 0.001
Year	2009	-0.8 (0.11)	0.45 (0.36–0.56)	< 0.001
	2011	-0.53 (0.11)	0.59 (0.47–0.73)	< 0.001
	2014	-1.27 (0.4)	0.28 (0.13–0.62)	0.002
Age		-0.01 (0.046)	0.99 (0.9–1.08)	0.788
Sex (male)		0.11 (0.089)	1.12 (0.94–1.33)	0.198
*Ascaris* (epg)		0.22 (0.055)	1.24 (1.12–1.39)	< 0.001
Hookworm (epg)		0.41 (0.075)	1.5 (1.3–1.74)	< 0.001
*Trichuris* (epg)		1.14 (0.084)	3.12 (2.65–3.68)	< 0.001
Random effects		Variance	SD	*P*
Pupil (Intercept)		0	0	1
School (Intercept)		0.56	0.75	

#### Prevalence of moderate and heavy infections

The majority of infections were found to be of light intensity. No more than five instances of heavy infection were detected during the course of the study in any species in either the pilot or extension schools; we therefore did not subject prevalence of heavy infection to analysis. Moderate infections were similarly sparse being below 0.2 % for *T. trichiura* and hookworm in all years, and mainly found in *A. lumbricoides* (Table 1). Models for the prevalence of infections which were at least moderate only converged for *A. lumbricoides* for extension schools (Table 8), and showed a non-significant decrease between baseline and 2011, and 2014, respectively (*P* = 0.097 and *P* = 0.759, respectively).

**Table 8:** Predictors for an infection with *Ascaris lumbricoides* of at least moderate intensity across all 19 extension study sentinel sites in Burundi combined with the intensity data obtained by a mapping survey in 2014 from 14 of those 19 schools. Results shown are from binomial mixed models based on *n* = 20,879 observations. Age had been mean centred and divided by the estimate of its standard deviation. The pseudo-*R* ^2^ values for this model were pseudo-*R* ^2^_MF_ = 0.195; *R* ^2^_marg._ = 0.078, *R* ^2^_cond._ = 0.662

Fixed effects	Category	Parameter	Adjusted odds ratio (CI)	*P*
(Intercept)		-5.92 (0.72)	0.0 (0–0.01)	< 0.001
Year	2009	-2.27 (1.2)	0.1 (0.01–1.15)	0.065
	2011	-1.23 (0.74)	0.29 (0.07–1.25)	0.097
	2014	-0.46 (1.5)	0.63 (0.03–11.83)	0.759
Age		-0.21 (0.082)	0.81 (0.69–0.95)	0.009
Age^2		-0.11 (0.075)	0.89 (0.77–1.04)	0.137
Sex (male)		0.16 (0.15)	1.18 (0.88–1.58)	0.282
Random effects		Variance	SD	*P*
Pupil (Intercept)		0.40	0.63	
School (Intercept)		5.30	2.30	
Year 2009		1.50	1.20	
Year 2011		0.82	0.91	
Year 2014		6.80	2.60	
Random Slope Year				< 0.001

#### Temporal changes in the spatial dependence of STH prevalence

**Fig. 3:**
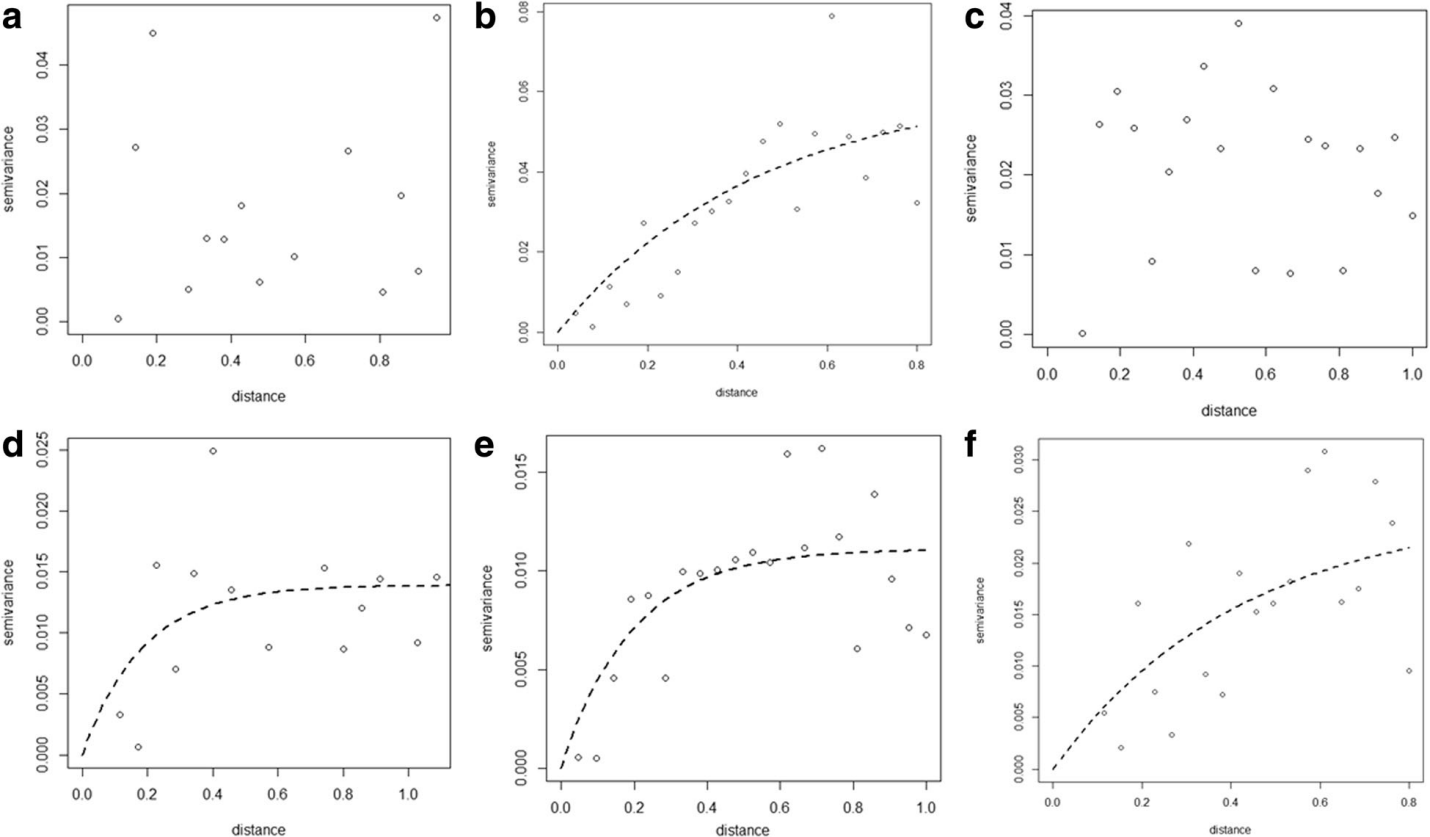
Yearly semivariograms for *Ascaris lumbricoides* (in decimal degrees). Each plot shows a semivariogram for each year of the impact study: **a** 2007, **b** 2008, **c** 2009, **d** 2010, **e** 2011, **f** 2014

**Fig. 4:**
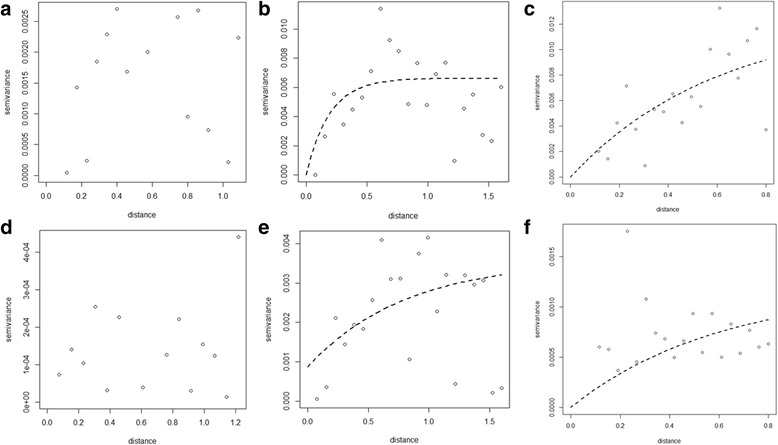
Yearly semivariograms for *Trichuris trichiura* (in decimal degrees). Each plot shows a semivariogram for each year of the impact study: **a** 2007, **b** 2008, **c** 2009, **d** 2010, **e** 2011, **f** 2014

**Fig. 5:**
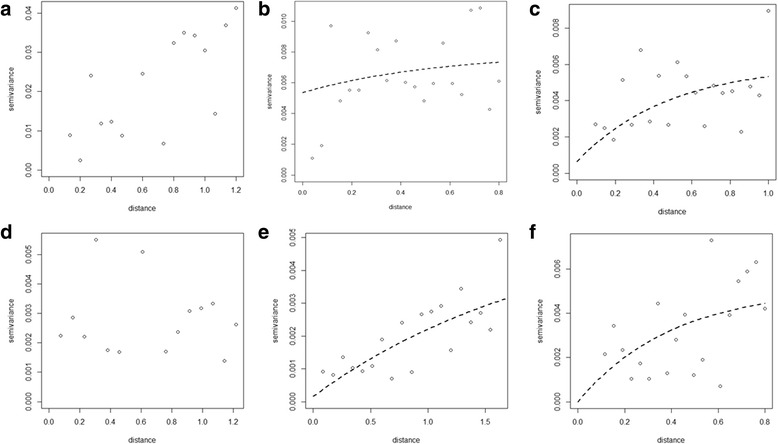
Yearly semivariograms for hookworm infection (in decimal degrees). Each plot shows a semivariogram for each year of the impact study: **a**2007, **b** 2008, **c** 2009, **d** 2010, **e** 2011, **f** 2014

Figures 3, 4 and 5 show semivariograms for the prevalence of each STH species in each year. Spatial dependence was evident for all parasite species, though not in every year of the study. Table 8 summarises these results. Our results show that prevalence of *A. lumbricoides* demonstrated spatial clustering in 2010 (Fig. 3d) and 2011 (Fig. 3e) with an average cluster size of 55 km and 101 km, respectively. A spatial trend in the prevalence of *A. lumbricoides* infection was evident in 2008 and 2014.

In the case of prevalence of *T. trichiura*, clustering was evident in 2008, and spatial trends in prevalence were evident in 2009, 2011 and 2014 (Fig. 4). No spatial dependency patterns were evident in 2007 and 2010 (Fig. 4). In the case of prevalence of hookworm infection (Fig. 5), our results showed no spatial clustering. However, our results indicate that a spatial trend in prevalence of hookworm infection was observed in 2008, 2009, 2011 and 2014 (Fig. 5).

#### Temporal changes in the spatial dependence of the prevalence of Ascaris lumbricoides intensity of infection profiles

Additional file 1: Figures S4 to S7 demonstrate the spatial variation in the prevalence of low intensity of infection profile for all other parasites. While there was no evidence of clustering in intensity of infection profiles for any of the STH species, results indicate a strong spatial trend in the prevalence of low intensity of infection category for *A. lumbricoides* (Additional file 1: Figure S4) in all years with the exception of 2007 and 2009. No spatial trends in the prevalence of moderate *A. lumbricoides* intensity of infection (Additional file 1: Figure S5) were evident in all years.

## Discussion

This study reports an epidemiological evaluation of the impact of 8 years of mass deworming in Burundi. Several impact surveys have been reported in the literature; however, apart from an 8-year follow up of schistosomiasis and intestinal helminthiasis in one school in Burundi [9], a study on 7 years of MDA in Myanmar [31] and a 2-year study on ALB/MBZ MDA deworming in Cuba [32], there is a significant lack of literature reporting repeated annual survey data evaluating the impact of long-term ALB/MBZ MDA on STHs both at national and district levels. There is also a significant gap in the literature addressing the long-term nationwide impact of an annual national MDA programme on the spatial heterogeneity of important direct infection indicators.

### Impact of MDA programme on STH infection indicators

Looking at the first 5 years of the NTD programme in Burundi, our data confirm that repeated mass administration of anthelmintic drugs significantly reduced the prevalence of STH single and co-infections in primary school children across Burundi. This significant reduction in infection prevalence was demonstrated in both pilot and extension studies from 2007 to 2011. However, there was a large amount of between-school variation in change of prevalence over time, as evidenced by the strong statistical significance of the school by year random interaction. This observation cannot be explained by the reported ALB/MBZ coverage data for these years in that the coverage of ALB/MBZ administration across the entire country was reported to be homogeneous with a satisfactory coverage, often over 95 % for school age children in each district [33]. An impact on intensity of infection could not be properly observed as the majority of infections were already within the low intensity category [34]; hence it was difficult to quantify any significant decrease in this parameter.

Along with this inter-school variance, a temporarily increased prevalence was observed in 2009 in a few specific schools in the pilot study. The first possible explanation could have been a low therapeutic coverage during the previous MDA round in those areas. However, the MoH reported that in December 2008, the coverage of ALB/MBZ treatment for school age children was above 88 % for pilot sites in Bururi, Bubanza and Cibitoke provinces. A possible explanation is that in 2009 a new training programme for technical ground staff was implemented. The introduction of this programme possibly improved the performance of laboratory technicians in some of these provinces, which may have increased parasite detection rates and thus influenced the prevalence (Ministry of Health statement, personal communication).

It is not clear whether inter-school variance and the temporary increase of prevalence could be attributed to changes of access to water and sanitation or other changes in environmental conditions. As mentioned in the methodology, observations related to the latrine status and access to water were collected just once during the period 2007–2011. The data reported presence of latrines in bad/mediocre conditions (over 90 % for pilot schools, and roughly 74 % for the extension schools), some access to water sources (28 and 16 % of pilot and extension schools with no access to water; other with access to water from rivers, tap and wells); and more than half of all the schools did not have a place where children could wash their hands. As these observations were not routinely reported, we cannot know whether there was any change in each school that could improve or worsen hygiene conditions. In general, during the period under study, according to the World Bank, between 73.5 and 79.5 % of the population in the country had access to improved water sources from year 2008 to year 2014; however, less than half of the population have access to sanitation facilities, with 45.9 % estimated in 2006 and 48.0 % reported in 2014, indicating an absence of significant changes of access to water and sanitation during the period under study, that could have had an impact on burden infection.

The analysis of data collected in the same schools in 2014 provided interesting insight into the long-term sustainability of MDA programmes. Despite six more cycles of ALB/MBZ treatments (delivered in June and December each year from 2011 to May 2014 during the MCHW), *A. lumbricoides* infection remained prevalent in the country. Raw data showed the absence of further prevalence reduction, and statistical models comparing the *A. lumbricoides* prevalence in 2014 with the one in 2007 in the pilot schools, demonstrated that there was no statistically significant decrease of infection since 2007. In the extension schools, the prevalence of *A. lumbricoides* dropped from 2008 to 2011, but no further decrease was observed from 2011 to 2014. Our results corroborate findings from other STH prediction models [27, 28, 35–37] which show that in areas with moderate baseline prevalence levels (as observed in this study), biannual treatment of school-age children can reduce the prevalence to the levels observed in Burundi in 2011 and 2014. However, after this initial reduction, no further reductions in prevalence are likely to be achieved. Hence, although MDA programmes are a viable option for morbidity control, they are less suitable as a sole intervention for reaching elimination goals [38]. It should also be taken into account that throughout these years, the onchocerciasis programme targeting the whole population with ivermectin and integrating treatment with either ALB or MBZ for treatment of worm infections in adults, was also running in ten districts (Cibitoke, Mabayi, Bubanza, Mpanda, Bururi, Matana Rumonge, Makamba, Rutana and Gihofi), where four the extension schools and six of the pilot schools were located (Ministry of Health onchocerciasis statements and reports to donors). It would have been expected that although children in the schools located in the onchocerciasis areas were not given a third annual treatment, treatment of the adult population could have somehow helped in decreasing infection in the population in those areas; however that was not, unfortunately, observed. Furthermore, political unrest and civil problems in Burundi and the resulting internal displacement of people was particularly intense in late 2009 and throughout 2010. This unrest most likely hindered the implementation of the epidemiological assessment in year 2010, and possibly also drug delivery in those schools where assessment was not feasible, and possibly in their catchment areas.

### Impact of MDA on spatial heterogeneity of STH infections

Investigation into the spatial variation in STH infections in the African context have previously been reported by others [36, 39] but previous studies failed to investigate the longitudinal effect of a recurring MDA programme on the spatial distribution of these infections such as the propensity for clustering and the average size of clusters. The novelty of this study lies in understanding the spatial heterogeneity in infection patterns, which has the potential to support targeted planning and strategy development prior to the implementation of the MDA programme. Our results provide new insights on how spatial dependence varied over time. The nature of this clustering also depends on the transmission domains of the various species [40], with *A. lumbricoides* and *T. trichiura* prevailing in the domestic domain and transmission/contamination occurring within or near the home and school environment, and hookworm prevailing in the public domain and thus more difficult to control.

Furthermore, the observed changes in the spatial dependence of STH infections, namely the transition from a clustered effect to a spatial trend in prevalence for *A. lumbricoides* and the fluctuations of spatial dependence in *T. trichiura* and hookworm parasites can be partially attributed to the success and effectiveness of MDA programmes in controlling STH infections. This may be because there is an underlying trend with local heterogeneity, and the MDA programme dampened the local clustering revealing this trend. Other contributing factors include the biology of the STH species. Hookworm larval stages ensure the parasites’ survival in the soil because their mobility means that this species may be better at adapting to changing environmental conditions. Thus, hookworm infections tend to be more widespread and transmission tends to occur in the public domain [40, 41]; on the contrary, for *A. lumbricoides* and *T. trichiura* prevalence, some spatial correlation is expected as both *A. lumbricoides* and *T. trichiura* exist in an egg form in the soil and unlike hookworm, they are immobile. This results in a more clustered effect since most infections with these parasites are more likely to occur in the household domain [40–42]*.* The spatial patterns for the intensity of infection followed a very similar trajectory as that of prevalence of infection indicators, for which spatial trends were evident.

### Limitations

The presented work has some limitations. With the intention of assessing impact on all worm infections, collection of data from the adult population would have been extremely useful to understand the impact on this age group. Especially, for hookworm, as assessment of samples from an older population (> 20–25 years of age [43]) would have been more appropriate to evaluate the impact of drug treatment on infection intensity. However, the collection of a sample cohort from this population with sufficient statistical power was not logistically possible within the routine national M&E strategy for Burundi. This is because the monitoring of children in sentinel schools was part of the national monitoring programme, and assessment of adults would have been beyond the scope of the programme, making it difficult to identify an appropriate time to treat all adults as well as the children without significant disruptions to the workforce and livelihoods.

This body of research utilised raw data for the spatial dependence analysis; however, it is generally known that certain environmental factors such as surface temperature, vegetation, rainfall and access to water may influence the spatial and temporal distribution of STH infections [42]. Thus, fixed effect models which account for these variables may be necessary, thus highlighting the necessity for further research into better understanding this relationship.

Some consideration should also be given to the fact that routine treatment started in a small part of the country in 2007 and disruptions in 2010 to all extension sites may have influenced the results. However, it is also important to consider the significance of the impact of this single missed year of treatments which resulted in the resurgence of infection prevalence to nearly baseline levels in some regions. This finding highlights the need for more enduring interventions which address the underlying issues associated with the proliferation of these parasites such as access to water and sanitation infrastructure and long-term health education interventions to be conducted concurrently with routine deworming programmes [1, 3, 44], if permanent reductions are to be sustained.

## Conclusions

Evaluation of the treatment impact has highlighted the importance of careful consideration of several important factors for sustained STH control. Two very significant results require attention: the first is the substantial reduction in moderate and high intensity infections from baseline to virtually non-existent levels in 2014; the second is the near elimination of co-infections from baseline to 2014. This shift in the intensity of infection profile and the prevalence of co-infections demonstrates the strong impact that a nationwide MDA programme can have even when implemented as a sole measure. Thus, our results reconfirm that mass treatment programmes are an effective control strategy yet not sufficient for elimination purposes, if not accompanied by other strategies aimed at improving access to clean water and sanitation, and incorporating social interventions (health education). Finally, further research on the impact on STH burden of increased anthelmintic treatment frequency and optimal target population is warranted if STH elimination is to be a priority. Increased treatment frequency (possibly up to four times a year), of a much wider population could possibly further reduce infection levels. In this case, monitoring drug efficacy should be an essential component of this research [45, 46].

## Additional file

Additional file 1:
**Table S1.** Sample sizes and numbers of pupils followed up by year for the impact study conducted in Burundi from 2007 to 2011. **Figure S1.** Semivariograms representing the prevalence of *Ascaris lumbricoides* and *Trichuris trichiura* coinfection from 2007 to 2011. **Figure S2.** Variation in temporal patterns of prevalence change across pilot study sentinel sites in Burundi between 2007 and 2011, and in 2014 based on the models presented in Table 4. **Figure S3.** Variation in temporal patterns of prevalence change across extension study sentinel sites in Burundi between 2007 and 2011, and in 2014 based on the models presented in Table 5. **Figure S4.** Semivariograms representing the prevalence of low infection intensity from 2007 to 2014 for *Ascaris lumbricoides*. **Figure S5.** Semivariograms representing the prevalence of moderate infection intensity from 2007 to 2014 for *Ascaris lumbricoides*. **Figure S6.** Semivariograms representing the prevalence of low infection intensity from 2007 to 2014 for *Trichuris trichiura*. **Figure S7.** Semivariograms representing the prevalence of low infection intensity from 2007 to 2014 for hookworm. (DOCX 3548 kb)

## Abbreviations

ALB: Albendazole
DALY: Disability adjusted life years
GLMM: Generalized linear mixed model
MBZ: Mebendazole
MDA: Mass drug administration
M&E: Monitoring and evaluation
MoH: Ministry of Health
NTD: Neglected tropical disease
SCI: Schistosomiasis Control Initiative
SCORE: Schistosomiasis Consortium for Operational Research and Evaluation
STH: Soil-transmitted helminthiasis
WHO: World Health Organization
