# Supplementary material for: The impact of an 8-year mass drug administration programme on prevalence, intensity and co-infections of soil-transmitted helminthiases in Burundi
Source: Parasit Vectors. 2016 Sep 22;9:513. doi: 10.1186/s13071-016-1794-9 (PMC5034474; doi:10.1186/s13071-016-1794-9)
Supplement: Additional file 1: — Table S1. Sample sizes and numbers of pupils followed up by year for the impact study conducted in Burundi from 2007 to 2011. Figure S1. Semivariograms representing the prevalence of Ascaris lumbricoides and Trichuris trichiura coinfection from 2007 to 2011. Figure S2. Variation in temporal patterns of prevalence change across pilot study sentinel sites in Burundi between 2007 and 2011, and in 2014 based on the models presented in Table 4. Figure S3. Variation in temporal patterns of prevalence change across extension study sentinel sites in Burundi between 2007 and 2011, and in 2014 based on the models presented in Table 5. Figure S4. Semivariograms representing the prevalence of low infection intensity from 2007 to 2014 for Ascaris lumbricoides. Figure S5. Semivariograms representing the prevalence of moderate infection intensity from 2007 to 2014 for Ascaris lumbricoides. Figure S6. Semivariograms representing the prevalence of low infection intensity from 2007 to 2014 for Trichuris trichiura. Figure S7. Semivariograms representing the prevalence of low infection intensity from 2007 to 2014 for hookworm. (DOCX 3548 kb) [file 13071_2016_1794_MOESM1_ESM.docx]

# **Additional File 1. Supplementary Tables and Figures**

# **The impact of an 8-year mass drug administration programme on prevalence, intensity and co-infections of soil-transmitted helminthiases in Burundi.**

# Giuseppina Ortu^1*^_,_ Mohamad Assoum^2, 3*^, Udo Wittmann^1^, Sarah Knowles^1^, Michelle Clements^1^, Onésime Ndayishimiye^4^, Maria-Gloria Basáñez^1, 5^, Colleen Lau^3, 6^, Archie Clements^6^, Alan Fenwick^1^, Ricardo J. Soares Magalhaes^3, 7^

^1^ Schistosomiasis Control Initiative, Imperial College London, Department of Infectious Disease Epidemiology, School of Public Health, Faculty of Medicine (St. Mary’s Campus), Norfolk Place, London W2 1PG, UK

^2^ School of Medicine, The University of Queensland, Brisbane, Australia

^3^ Children’s Health and Environment Program, Child Health Research Centre, The University of Queensland, Brisbane, Australia.

^4^ Programme National Intégré de lutte contre les Maladies Tropicales Négligées et la Cécité (PNIMTNC) - Ministère de la Santé Publique et de la lutte contre le SIDA, Burundi

^5^ London Centre for Neglected Tropical Disease Research, Imperial College London, Department of Infectious Disease Epidemiology, School of Public Health, Faculty of Medicine (St. Mary’s Campus), Norfolk Place, London W2 1PG, UK

^6^ Research School of Population Health, Australian National University, Canberra, Australia.

^7^School of Veterinary Science, The University of Queensland (Gatton Campus), Via Warrego Highway, Gatton QLD 4343, Australia

** Contributed equally to this work*

**Corresponding author:**

Dr. Giuseppina Ortu, email: [g.ortu@malariaconsortium.org](mailto:g.ortu@malariaconsortium.org)

**Supplementary Tables**

Table S1: **Sample sizes and numbers of pupils followed up by year for the impact study conducted in Burundi from 2007 to 2011.**

| Follow up year | Year when cohort entered the study | | | | |
| --- | --- | --- | --- | --- | --- |
| **Pilot Study** | **2007** | **2008** | **2009** | **2010** | **2011** |
| 2007 | 3616 | 0 | 0 | 0 | 0 |
| 2008 | 1188 | 2288 | 0 | 0 | 0 |
| 2009 | 1769 | 864 | 2311 | 0 | 0 |
| 2010 | 1326 | 682 | 830 | 2224 | 0 |
| 2011 | 1276 | 660 | 726 | 816 | 1202 |
|  |  |  |  |  |  |
| **Extension Study** | **2007** | **2008** | **2009** | **2010** | **2011** |
| 2008 |  | 5700 | 0 |  | 0 |
| 2009 |  | 3047 | 3331 |  | 0 |
| 2011 |  | 2158 | 1124 |  | 5587 |

## **Supplementary Figures**

Figure S1: **Semivariograms representing the prevalence of** ***Ascaris lumbricoides* and *Trichuris trichiura* coinfection from 2007 to 2011.**


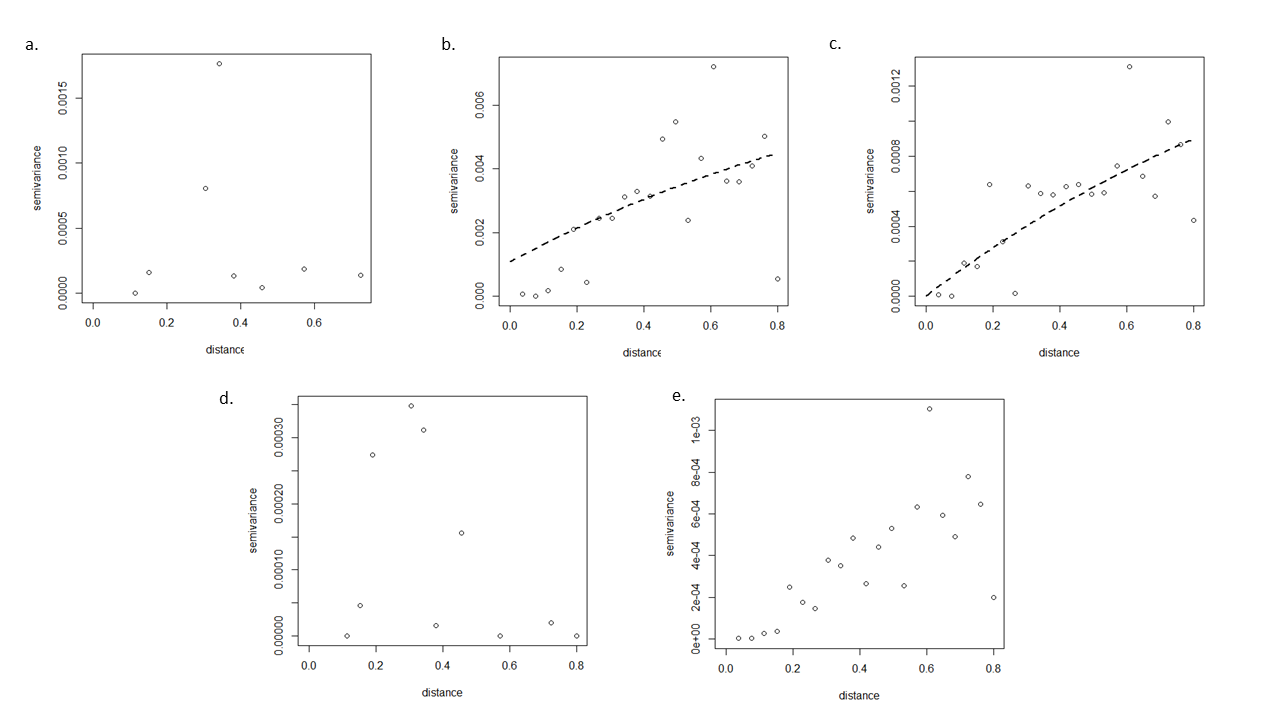


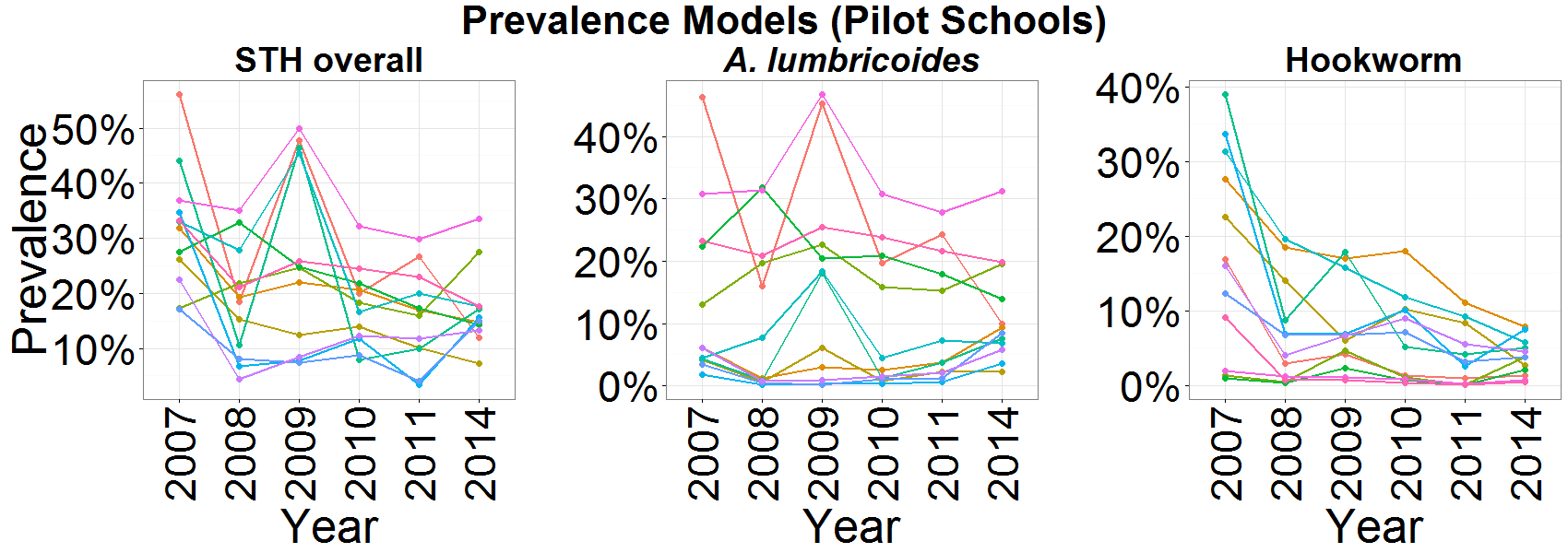
Figure S2: **Variation in temporal patterns of prevalence change across pilot study sentinel sites in Burundi between 2007 and 2011, and in 2014 based on the models presented in Table 4 of the main text. Each coloured line represents the infection prevalence at a different sentinel site as described by the model**

**
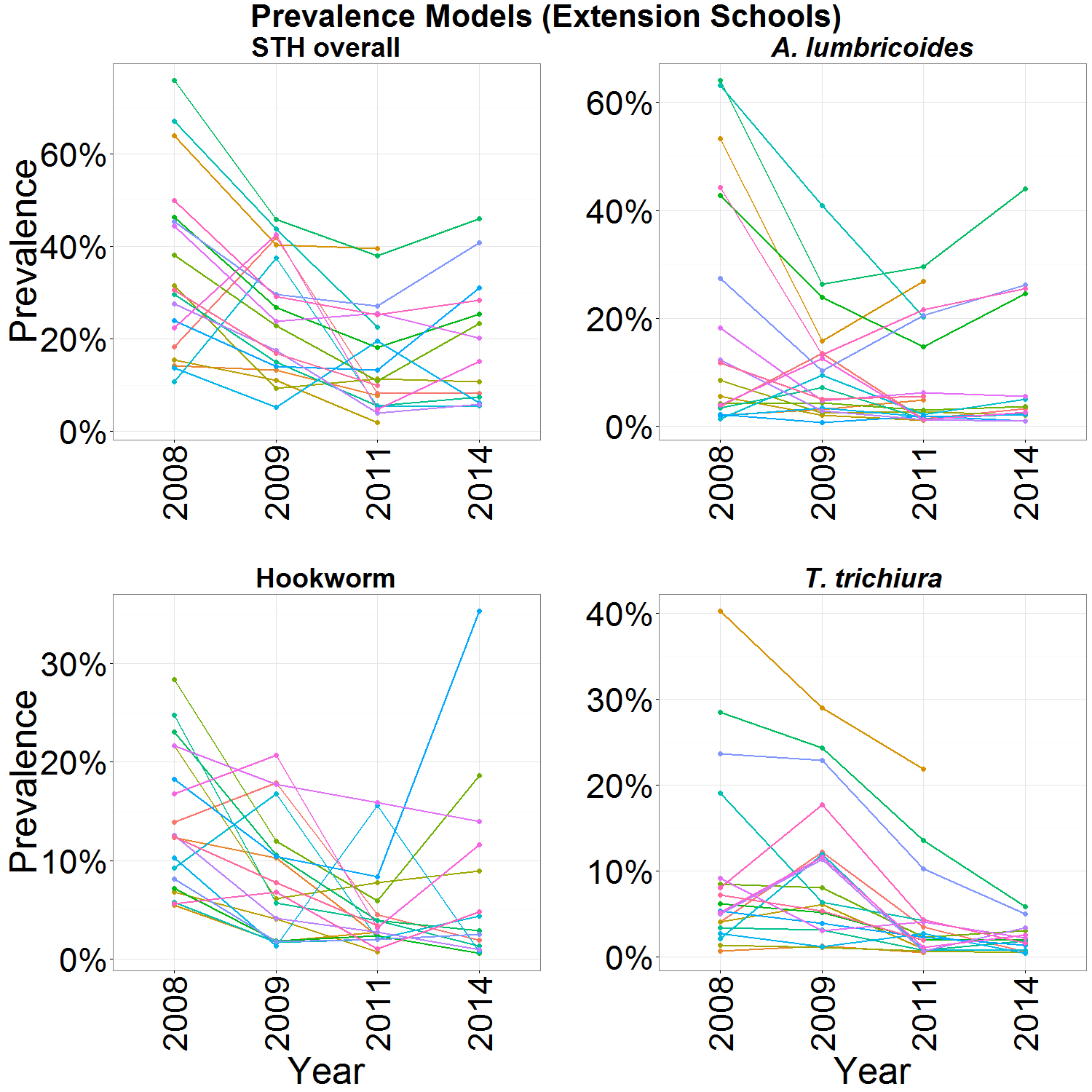
**Figure S3: **Variation in temporal patterns of prevalence change across extension study sentinel sites in Burundi between 2007 and 2011, and in 2014 based on the models presented in Table 5 of the main text. Each coloured line represents the infection prevalence at a different sentinel site as described by the model. Five schools did not submit STH data for the mapping survey in 2014.**

Figure S4: **Semivariograms representing the prevalence of low infection intensity from 2007 to 2014 for *Ascaris lumbricoides*.**


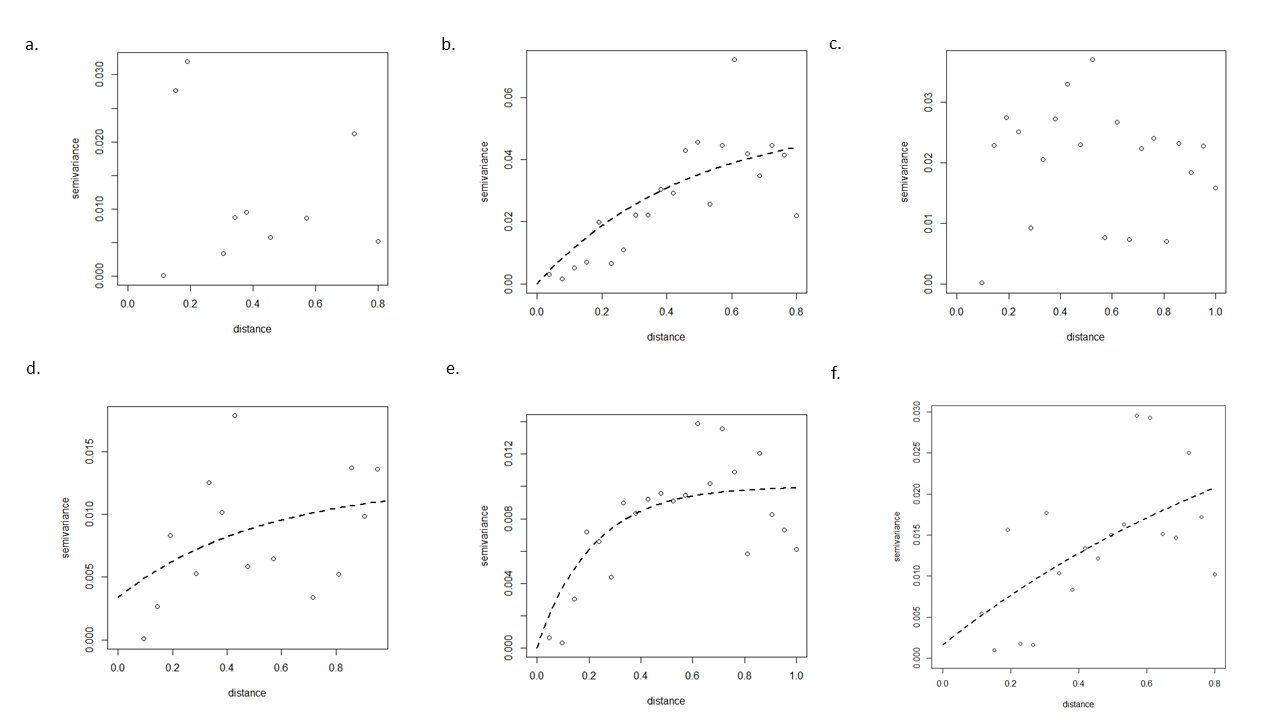


Figure S5: **Semivariograms representing the prevalence of moderate infection intensity from 2007 to 2014 for *Ascaris lumbricoides*.**


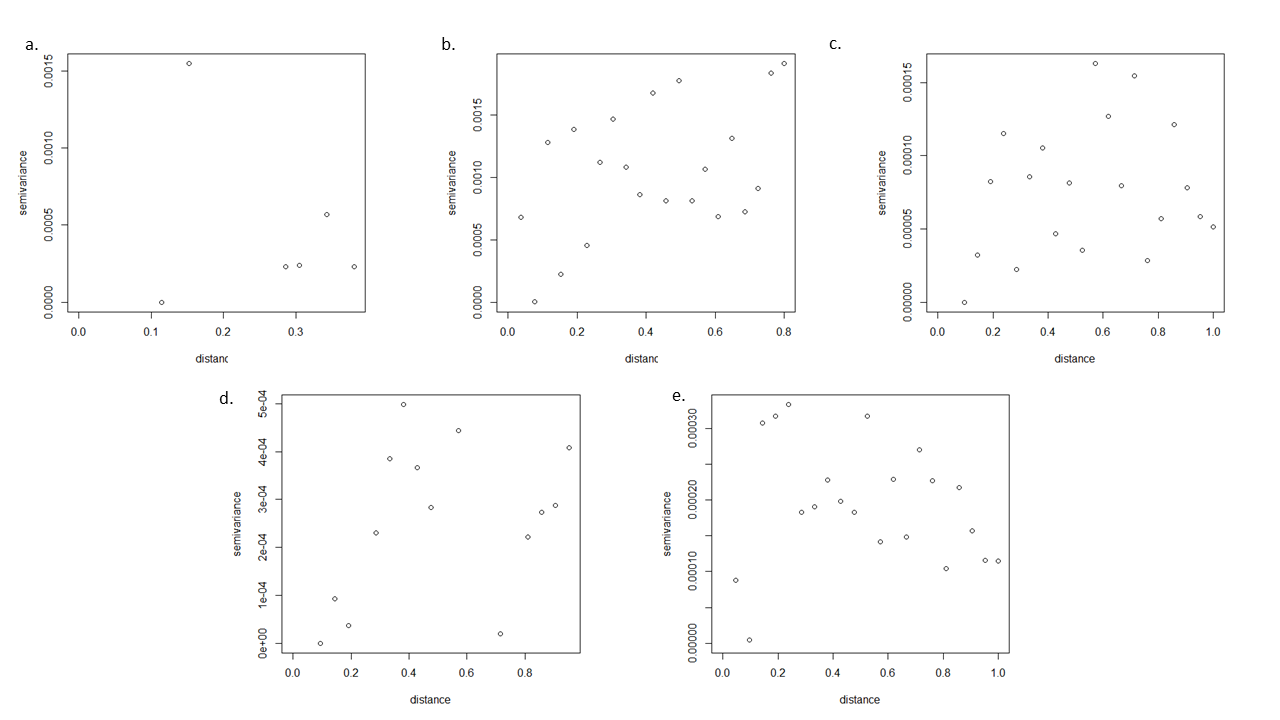


Figure S6: **Semivariograms representing the prevalence of low infection intensity from 2007 to 2014 for *Trichuris trichiura*.**


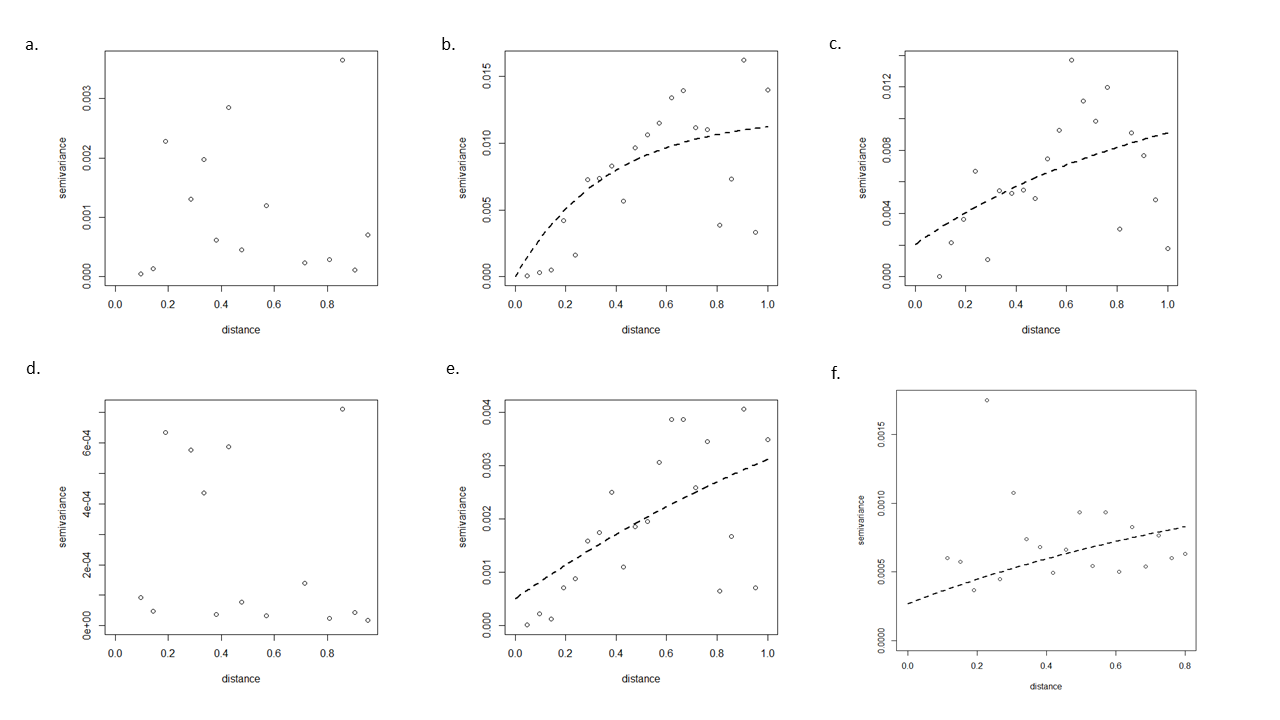


Figure S7: **Semivariograms representing the prevalence of low infection intensity from 2007 to 2014 for hookworm.**

**
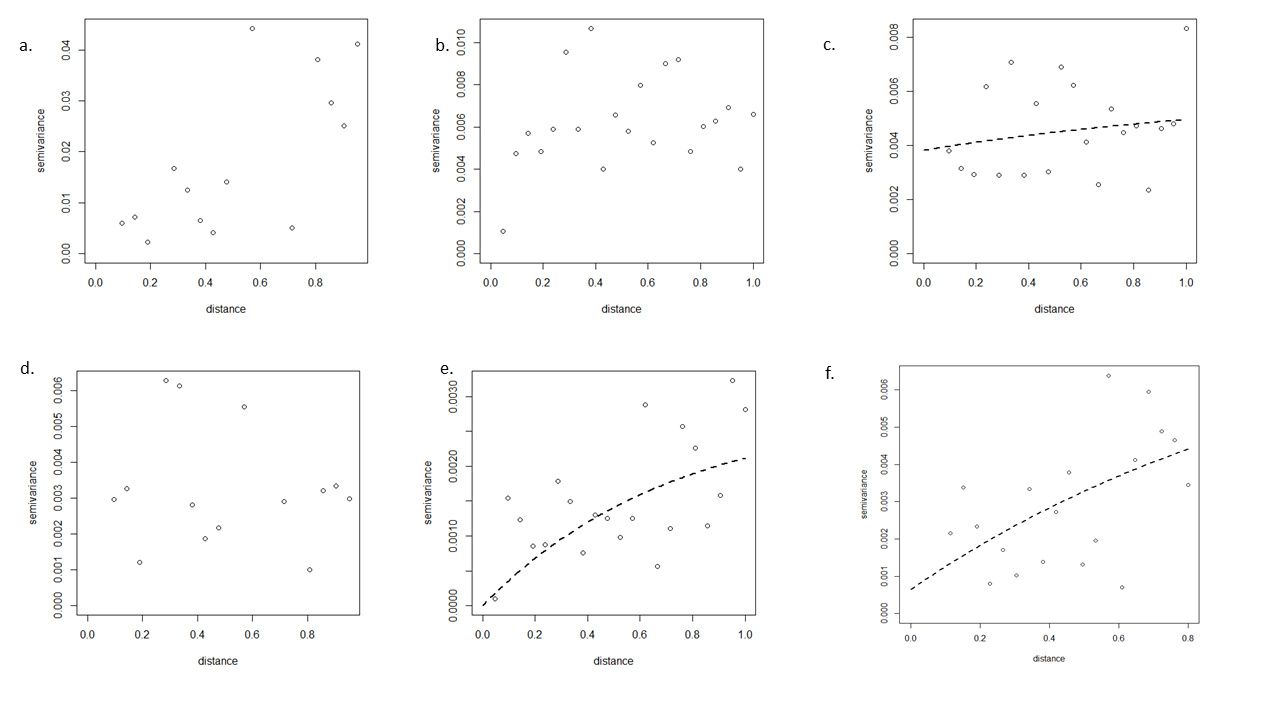
**
